# Imagery Rescripting for Pathological Affective Dependence in Intimate Partner Violence (ImRs‐PAD): An In‐Person Proof‐of‐Concept Study

**DOI:** 10.1002/cpp.70339

**Published:** 2026-09-24

**Authors:** Erica Pugliese, Allison Uvelli, Maria Grazia Foschino Barbaro, Carolina Papa, Federica Visco‐Comandini, Francesco Mancini, Arnold A. P. van Emmerik

**Affiliations:** ^1^ Department of Clinical Psychology University of Amsterdam Amsterdam the Netherlands; ^2^ Associazione di Psicologia Cognitiva (APC) and Scuola di Psicoterapia Cognitiva (SPC) Rome Italy; ^3^ Department of Medical Science, Surgery and Neurosciences University of Siena Siena Italy; ^4^ Associazione Italiana di Psicoterapia Cognitiva (AIPC) Bari Italy; ^5^ Department of Psychology Sapienza University of Rome Rome Italy; ^6^ Department of Human Sciences Guglielmo Marconi University Rome Italy

**Keywords:** imagery rescripting, intimate partner violence, pathological affective dependence, psychological intervention, relational entrapment, trauma‐related processes

## Abstract

This study evaluated the feasibility and preliminary effects of in‐person Imagery Rescripting for Pathological Affective Dependence (ImRs‐PAD), a trauma‐focused and mechanism‐based intervention for survivors of intimate partner violence (IPV). ImRs‐PAD targets trauma‐related relational dynamics—specifically Pathological Affective Dependence (PAD)—together with the related relational processes that may contribute to difficulties disengaging from abusive relationships. PAD is conceptualized as a relational constraint rooted in trauma, rather than individual pathology or responsibility. This study employed an exploratory single‐arm repeated‐measures proof‐of‐concept design conducted in Italy. Eighteen adult women survivors of IPV received a 13‐session ImRs‐PAD intervention, delivered within anti‐violence services by supervised psychotherapy trainees. Primary outcomes included state and trait levels of PAD, relational commitment and intention to return to the abusive relationship, which were conceived as different, although potentially overlapping self‐report indicators of relational entrapment. Secondary outcomes assessed complex trauma symptoms, psychological well‐being and resilience. Participants were assessed at baseline/pre‐treatment, mid‐treatment, post‐treatment and 1‐month follow‐up. Significant reductions were observed in PAD, relational commitment and intention to return over the course of the intervention. Improvements were also noted in trauma‐related symptoms, psychological well‐being and resilience from baseline to post‐treatment. At 1‐month follow‐up, changes in the primary relational outcomes and resilience were largely maintained, whereas trauma‐related symptoms showed slight attenuation but remained significantly improved relative to baseline. ImRs‐PAD appears to be a feasible and promising intervention in real‐world anti‐violence centre settings, specifically targeting unmet relational needs implicated in IPV‐related entrapment. Changes in the primary relational outcomes were more consistently maintained at follow‐up than improvements in trauma‐related symptoms after treatment termination, suggesting the potential importance of integrating such interventions within broader systems of psychosocial support.

**Trial Registration:** ClinicalTrials.gov identifier: NCT06670326

## Summary

- Pathological Affective Dependence (PAD) represents a trauma‐related relational mechanism that contributes to difficulties disengaging from abusive relationships in intimate partner violence (IPV).
- Imagery Rescripting adapted to target PAD (ImRs‐PAD) appears feasible when delivered in person with real‐world clinical cases recruited through anti‐violence services.
- Targeting unmet relational needs may help reduce PAD‐related relational entrapment, including internal conflict, perceived inability to separate and commitment to the abusive relationship.
- Changes in the primary relational outcomes appeared to be more consistently maintained than improvements in symptom‐level outcomes, suggesting the potential value of integrating interventions within broader psychosocial support systems.

## 1 Introduction

Intimate partner violence (IPV) is a global public health issue with far‐reaching consequences for both physical and mental health. IPV refers to a multifaceted pattern of abuse occurring within current or former intimate relationships, encompassing psychological abuse, coercive control, physical violence and sexual violence, with marked variability in severity, chronicity and relational dynamics (World Health Organization [WHO] 2021). Chronic exposure to interpersonal trauma has been consistently linked to increased vulnerability to depression, anxiety, post‐traumatic stress disorder (PTSD), complex PTSD (CPTSD) and somatic symptoms such as chronic pain (Hameed et al. 2020; Uvelli et al. 2024).

According to the WHO, nearly one in three women worldwide has experienced physical and/or sexual IPV in her lifetime—a prevalence that has remained alarmingly stable over the past two decades (WHO 2021). The Global Burden of Disease Study estimates that IPV contributes substantially to years lived with disability (DALYs), through both psychological and physical sequelae (Jewkes and Abrahams 2026). Italian data reflect similar patterns of prevalence and impact (ISTAT 2025). Research has increasingly pointed to the role of early relational trauma and childhood maltreatment as key developmental risk factors for IPV victimization in adulthood (Capaldi et al. 2012; Pugliese et al. 2024; Widom et al. 2015).

Despite this growing recognition, most interventions for IPV survivors continue to focus on safety planning, symptom reduction or empowerment strategies, with inconsistent effects on trauma‐related symptoms, self‐efficacy and risk of revictimization (Hameed et al. 2020).

As highlighted by Oram et al. (2022), there is an urgent need for treatment models that more effectively integrate trauma history with relational and contextual mechanisms influencing recovery trajectories in IPV. In this light, trauma‐focused interventions specifically tailored to address relational vulnerabilities remain scarce and fragmented, despite their potential to promote deeper and more sustainable recovery. IPV‐related psychopathology may be shaped and maintained by a range of post‐traumatic and affective processes, including emotional dysregulation, dissociation, shame, guilt, alexithymia and suicidal ideation (Veggi et al. 2024; Torrioni et al. 2025). These processes may function as transdiagnostic mechanisms through which early traumatic experiences increase vulnerability to later interpersonal difficulties. In particular, they may undermine self‐protection, boundary‐setting and the ability to disengage from harmful relationships, thereby contributing to victimization‐related interpersonal problems and persistent psychological distress (Visco‐Comandini et al. 2026). Within this framework, pathological affective dependence (PAD) has emerged as a clinically relevant but under‐addressed trauma‐related relational vulnerability. PAD is theoretically grounded in the Cognitive Model of PAD proposed by Pugliese et al. (2023), which conceptualizes PAD as the result of early interpersonal trauma and the chronic frustration of fundamental relational needs—including love, dignity and safety (Speranza et al. 2022; Stith et al. 2000). These unmet needs contribute to maladaptive relational schemas that reinforce dependency, fear of abandonment and relational entrapment. Clinically, PAD manifests through (1) a pervasive internal conflict between the impulse to separate and the need to preserve the relationship; (2) suffering of ongoing abuse; and (3) a perceived inability to disengage, even in the face of escalating harm (Pugliese 2024; Pugliese 2026; Pugliese et al. 2025). Actual separation is particularly destabilizing, often characterized by intense emotional distress and repeated cycles of rupture and reconciliation. Importantly, this phase also represents the period of greatest risk for severe injury or femicide (Bell et al. 2007), underscoring the critical need for targeted interventions that address the relational mechanisms maintaining risk exposure. PAD should therefore be distinguished from trauma bonding, general attachment insecurity and broader relational vulnerability frameworks. Trauma bonding explains how an emotional bond may develop or be reinforced within cycles of abuse, fear, coercive control, reconciliation, intermittent reinforcement and power imbalance (Reid and Gardy 2024; Isaiah et al. 2024). Attachment insecurity, in contrast, describes a broader relational vulnerability, including fear of abandonment, heightened proximity seeking, avoidance of intimacy, affect dysregulation and negative expectations about the self and others, which may increase vulnerability to IPV but does not specifically explain why separation becomes subjectively impossible (Velotti et al. 2018; Spencer et al. 2024). PAD partially overlaps with these constructs, but it differs from them because it specifically captures why the person remains psychologically stuck in the relationship: not only because she is emotionally attached, insecure or traumatized but also because separation is experienced as emotionally intolerable or impossible, despite suffering and awareness of the harmful nature of the relationship. In this sense, attachment insecurity may represent a distal vulnerability factor for PAD, and trauma bonding may describe one relational pathway through which PAD is reinforced, whereas PAD identifies the more proximal clinical mechanism maintaining commitment to a dysfunctional or abusive relationship.

Conceptually, PAD operates along both trait‐like dimensions—as a chronic relational pattern—and state‐like dynamics, wherein acute emotional dysregulation is triggered by perceived threats to the attachment bond, particularly during phases of separation. The recent development of the PAD scale (Pathological Affective Dependence Scale [PADS]) (Pugliese et al. 2025) allows for systematic assessment of both trait and state aspects of PAD. However, to date, evidence on interventions specifically targeting PAD in survivors of IPV remains very limited, and no controlled studies have yet evaluated PAD‐focused interventions in this population. Given its focus on emotionally charged autobiographical memories, unmet developmental needs and the transformation of trauma‐related meanings, imagery rescripting (ImRs) may represent a particularly suitable mechanism‐based intervention for PAD‐related relational entrapment (Visco‐Comandini et al. 2025). ImRs is a well‐established experiential technique that targets trauma‐related vulnerability through the transformation of distressing autobiographical memories. Originally developed within the framework of Schema Therapy, ImRs has since been adapted for use in cognitive behavioural therapy and other evidence‐based modalities (Arntz and van Genderen 2020; Schaich et al. 2020). The intervention works by reactivating emotionally charged early memories and modifying their meaning through guided imagery, often involving the symbolic fulfilment of unmet developmental needs (Müller‐Engelmann and Steil 2017; Koetsier et al. 2024).

This mechanism is particularly relevant for PAD, which is conceptualized as involving the chronic frustration of core relational needs, including love, dignity and safety. By experientially addressing these unmet needs within autobiographical memories, ImRs may target the emotional meanings that sustain relational entrapment, such as helplessness, unworthiness, fear of abandonment and perceived inability to separate. In this sense, ImRs is expected to affect PAD‐related mechanisms not only through general trauma symptom reduction but also through processes of unmet‐need fulfilment, mastery, emotional meaning updating and counterconditioning (Arntz 2014; Koetsier et al. 2024; Quinton et al. 2018; Rameckers et al. 2024). A growing body of clinical trials and meta‐analyses supports its efficacy in treating trauma‐related disorders, particularly PTSD following childhood abuse. Outcomes appear comparable to those achieved by established first‐line treatments, including trauma‐focused cognitive behavioural therapy and EMDR (De Haan et al. 2020; Morina et al. 2017; Raabe et al. 2022; Visco‐Comandini et al. 2025). Preliminary findings from a multiple case series study involving three adult female survivors of IPV, who completed an individual online Imagery Rescripting for Pathological Affective Dependence (ImRs‐PAD) protocol, showed promising clinical changes. These included reductions in depression, anxiety, somatic symptoms, PTSD‐related symptoms and state‐level PAD, as well as increases in psychological well‐being, resilience and self‐compassion (Pugliese et al. 2026). While preliminary, these findings from the previous online multiple case series support the clinical utility and feasibility of ImRs‐PAD as a mechanism‐focused intervention for relational entrapment in IPV.

### 1.1 Rationale

Within the framework of complex trauma, the PAD model offers a robust theoretical rationale for applying ImRs to survivors of IPV. According to this model, when core interpersonal needs such as love, dignity and safety are chronically frustrated in early caregiving environments, individuals may develop PAD. In this framework, love does not simply refer to romantic affection but to the basic emotional experience of being worthy of care, being allowed to prioritize oneself and not having to organize one's identity around the excessive caregiving of a fragile, problematic or abusive other. This form of developmental vulnerability predisposes them to relational difficulties in adult intimate partnerships (Pugliese et al. 2023). In such relationships, unresolved relational schemas are often reactivated under conditions of interpersonal threat or abandonment, contributing to affective entrapment and difficulty disengaging from abusive partners. ImRs‐PAD was therefore selected because its central therapeutic mechanism directly corresponds to this theoretical model: it aims to experientially address unmet emotional needs and transform the autobiographical meanings associated with them. By targeting these unmet needs, ImRs‐PAD may modify the emotional meanings that sustain PAD‐related relational entrapment, including helplessness, unworthiness, fear of abandonment, excessive responsibility for the partner and perceived inability to separate. In the present study, these processes were operationalized through conceptually related but distinct self‐report indicators, including state and trait PAD, relational commitment and intention to return. Although ImRs has demonstrated efficacy in the treatment of trauma‐related disorders, it has not yet been systematically tested in IPV populations. Furthermore, it has never been specifically applied to the trauma‐related relational vulnerabilities implicated in PAD.

### 1.2 Objectives

The study adopts a trauma‐informed framework in which vulnerability to revictimization is not regarded as the result of personal weakness or poor judgement. Instead, it is understood as the product of trauma‐related psychological mechanisms, relational constraints and structural or contextual barriers that undermine the ability to disengage from abusive dynamics (Gordon et al. 2004; Rusbult et al. 1998). Accordingly, the primary objectives of this study were to evaluate whether ImRs‐PAD is associated with measurable changes in PAD (state and trait), relational commitment and intention to return to the abusive partner. These constructs were conceptualized as overlapping but distinct manifestations of relational entrapment: State PAD reflects transient relational dependency, trait PAD reflects more stable dependency patterns, relational commitment captures psychological investment in the relationship, and intention to return reflects motivational orientation towards reunification with the abusive partner. Secondary objectives of the study were to explore the impact of ImRs‐PAD on trauma‐related symptoms, psychological well‐being and resilience. Psychological well‐being was conceptualized as a broad subjective appraisal of positive emotional and functional health, including positive affect, vitality, self‐control and perceived general health, rather than merely the absence of psychological symptoms (Grossi et al. 2006). Resilience was defined more specifically as the perceived ability to recover or ‘bounce back’ from stress, consistent with its operationalization through the Brief Resilience Scale (BRS; Smith et al. 2008). More broadly, resilience is increasingly understood as a dynamic process shaped by individual, relational and contextual resources rather than as a fixed personal characteristic (Windle 2011). Given that IPV is associated with post‐traumatic stress, emotional dysregulation and impaired adaptive functioning, examining psychological well‐being and resilience alongside trauma‐related symptoms may provide a broader account of recovery and positive adaptation (Carannante et al. 2025).

Building on preliminary findings from the previous online multiple case series (Pugliese et al. 2026), the present study examined the same manualized ImRs‐PAD protocol delivered in person.

### 1.3 Hypotheses

In line with the primary objectives, we hypothesized that survivors of IPV would show significant within‐subject improvements from baseline to post‐treatment, with effects sustained at follow‐up (FU). Specifically, following the ImRs‐PAD intervention, participants were expected to show reductions in both state‐level PAD (PAD‐S) and trait‐level PAD (PAD‐T), relational commitment, and intention to return to the abusive partner, with these reductions maintained or further reduced at 1‐month FU. In line with the secondary objectives, participants were expected to report decreases in CPTSD symptoms, including both core post‐traumatic stress and disturbances in self‐organization (DSO), with either maintenance or further reduction at FU. Increases were expected in psychological well‐being and resilience from baseline to post‐treatment, with these gains either maintained or further improved at FU.

## 2 Method

### 2.1 Study Design

The present study employed an exploratory single‐arm repeated‐measures proof‐of‐concept design to examine the feasibility and preliminary effects of in‐person ImRs‐PAD. Eighteen participants were recruited from anti‐violence centres in Italy and assigned to randomly staggered baseline durations ranging from 5 to 9 weeks, in cohorts of approximately five participants each. This arrangement was originally intended to implement a non‐concurrent multiple‐baseline design. During the baseline phase, participants were scheduled to complete one assessment per week before treatment initiation. However, due to technical issues during data collection—specifically, a failure in the online questionnaire system—baseline data were incomplete for some participants, limiting the feasibility of the intended design. Of the 18 participants enrolled, only six completed the intended repeated‐baseline assessment schedule. Importantly, all six participants belonged to the same baseline‐duration group, preventing meaningful evaluation of staggered baseline effects across different baseline lengths. Because the remaining repeated‐baseline data were insufficient and lacked variability across baseline conditions, the originally planned multiple‐baseline analytic approach could not be implemented. Consequently, the final analytic design was an exploratory single‐arm repeated‐measures proof‐of‐concept study conducted at fixed time points: baseline/pre‐treatment, mid‐treatment, post‐treatment and 1‐month FU. Given the focus on feasibility and preliminary within‐subject signals of change, no control or comparison group was included. The broader ImRs‐PAD protocol was registered on ClinicalTrials.gov under the identifier NCT06670326. The present manuscript reports a preliminary proof‐of‐concept investigation based on the available repeated‐assessment data and should therefore be interpreted as an exploratory step preceding larger controlled evaluations of the intervention.

### 2.2 Participants

The sample included 18 adult women (M age = 47.22, SD = 11.01) referred by anti‐violence centres in Southern Italy collaborating with the research team. Access to these services was required to ensure integrated support such as safety planning and legal aid. Inclusion criteria were age ≥ 18, confirmed IPV history, completion of the PADS (state and trait) and informed consent. Exclusion criteria included intellectual disability, psychotic or bipolar disorders, clinically significant dissociative disorders and current psychotropic medication. Exclusion criteria were assessed through clinical interviews and information collected by anti‐violence centre staff regarding previous diagnoses and clinical history. Eligibility decisions were reached jointly by the assigned therapist, the clinical supervisor and anti‐violence centre professionals to ensure consistency and participant safety. All participants met eligibility criteria and completed the full treatment and assessment protocol without dropout.

### 2.3 Procedure

The study procedure consisted of six sequential phases. (1) Initial contact and informed consent: Participants initially accessed an anti‐violence centre, where the study was first presented in general terms. Next, more detailed information about study procedures was provided, and participants were given the opportunity to ask questions before providing written informed consent. This phase was managed by the anti‐violence centre and took place before the intervention began. (2) Eligibility assessment: Following consent, each participant was assigned to a trained therapist, who conducted a clinical evaluation to confirm eligibility based on the predefined inclusion and exclusion criteria. Phases 1 and 2 were completed within the same week. (3) Baseline assessment: During the baseline phase, initial data were collected without any therapeutic intervention. Participants completed the assessment questionnaires, and traumatic experiences related to unmet needs for love, dignity and safety were identified through clinical evaluation. In particular, three types of traumatic episodes were explored: (a) an episode of profound devaluation or humiliation in which the participant experienced feelings of worthlessness; (b) an episode of inverted attachment or parentification, in which the participant assumed a caregiving role towards an adult; and (c) an episode involving abandonment, neglect or physical abuse. (4) Psychoeducational intervention: Following the baseline phase, participants attended a single psychoeducational session. This session provided information about ImRs and its effectiveness in the treatment of trauma‐related disorders, as well as psychoeducation on the model of PAD. This psychoeducational session constituted Session 1 of the treatment. (5) Treatment phase: After completion of the psychoeducational session, participants entered the treatment phase (12 sessions), which lasted 12 weeks. The intervention consisted of ImRs sessions delivered by second‐ and third‐year post‐graduate psychotherapy trainees during their clinical internships. Six therapists delivered the intervention, each treating between one and four participants. All therapists received specific training by the first author in the ImRs‐PAD protocol before beginning treatment delivery. Supervision was provided in online group meetings held every 2 weeks by the first author, a senior clinician with expertise in trauma‐focused cognitive psychotherapy and ImRs. In addition, the first author remained available for individual meetings or contacts when specific clinical needs or difficulties emerged. Supervision focused primarily on the application of the ImRs‐PAD protocol and on difficulties encountered in conducting ImRs procedures. Intervention fidelity was supported through the use of a structured treatment protocol and regular supervision; however, fidelity was not formally assessed through independent ratings or session coding. Similarly, adherence to the protocol was monitored during supervision sessions but was not evaluated using a formal adherence checklist or standardized rating procedure. Risk management was conducted in collaboration with the referring anti‐violence centres, which remained available for safety planning, legal guidance and practical support when needed. Participant safety was monitored throughout the intervention through ongoing communication between trainees, the first author and anti‐violence centre staff. Therapists were instructed to report any indication of clinically significant deterioration, increased risk or adverse events to the first author, so that appropriate clinical or service‐based actions could be discussed and implemented. Outcome measures were administered again at mid‐treatment (after the sixth ImRs‐PAD session) and at the end of treatment (after the twelfth session). (6) FU assessment: An FU assessment was conducted 1 month after the final treatment session. Participants completed the assessment questionnaires again, without receiving any additional therapeutic intervention during the period between the end of treatment and the FU assessment.

### 2.4 Intervention: ImRs‐PAD Protocol

The ImRs‐PAD intervention consisted of 13 weekly individual sessions of approximately 50 min each, corresponding to Phases 4 and 5 of the study procedure described above. Specifically, the first session focused on providing psychoeducation about PAD and introducing the ImRs‐PAD protocol. The remaining 12 sessions involved active ImRs treatment. The protocol followed a past–present–future framework. Sessions 2–7 targeted early autobiographical memories related to the chronic frustration of core emotional needs, particularly love, dignity and safety. Sessions 8–10 focused on traumatic or emotionally significant memories involving the current or former abusive partner. Sessions 11–13 used future‐oriented imagery procedures to strengthen self‐protection, separation‐related mastery and relapse prevention. Across phases, PAD‐specific targets included perceived inability to separate, fear of abandonment, helplessness, unworthiness, excessive responsibility for the partner, difficulty prioritizing oneself and difficulty protecting one's own safety and dignity. The therapist initially took an active role in guiding the rescripting process, modelling protective and validating responses, providing emotional containment and helping the patient access unmet emotional needs. As treatment progressed, patients were gradually encouraged to assume a more active role in rescripting their experiences, thereby promoting autonomy, affect regulation, self‐protection and relational agency. A detailed session‐by‐session outline of the ImRs‐PAD protocol, including examples of imagery procedures, therapist instructions and PAD‐specific clinical targets, is provided in Appendix A. The intervention followed the same manualized ImRs‐PAD protocol described elsewhere in an online format (Pugliese et al. 2026; Pugliese 2024), whereas in the present study, sessions were delivered in person (see Table 1 for an overview of the protocol's description).

**TABLE 1:** Overview of the ImRs‐PAD intervention protocol.

Phase	Session	Focus
Psychoeducation and protocol introduction	Session 1	The first session focused on providing psychoeducation about PAD and introducing the ImRs‐PAD protocol.
Working on PAD past: Traumatic memories involving parents	Session 2	Therapist‐led ImRs: Need 1—Love
	Session 3	Therapist‐led ImRs: Need 2—Dignity
	Session 4	Therapist‐led ImRs: Need 3—Safety
	Session 5	Patient‐led ImRs: Need 1—Love
	Session 6	Patient‐led ImRs: Need 2—Dignity
	Session 7	Patient‐led ImRs: Need 3—Safety
Working on PAD present: Traumatic memories involving the partner or former partner	Session 8	Patient‐led ImRs: Need 1—Love
	Session 9	Patient‐led ImRs: Need 2—Dignity
	Session 10	Patient‐led ImRs: Need 3—Safety
Working on PAD future: Prevention and reduction of PAD relapse	Session 11	Patient‐led ImRs: Need 1—Love
	Session 12	Patient‐led ImRs: Need 2—Dignity
	Session 13	Patient‐led ImRs: Need 3—Safety

### 2.5 Measures

PAD was assessed using the PADS (Pugliese et al. 2025), a 17‐item self‐report questionnaire developed to assess maladaptive dependency patterns within intimate relationships. The instrument is available in two parallel forms measuring state (PADS‐S) and trait (PADS‐T) pathological affective dependence. The scale comprises three dimensions: Internal Conflict, reflecting emotional ambivalence and subjective distress related to the relationship (e.g., ‘I am aware that I am suffering in this relationship, but at the same time, I cannot leave it’); Inability to Separate from the Partner, indexing difficulties in disengaging from the partner despite negative relational experiences (e.g., ‘I would do anything in order not to lose my importance in my partner's sight’); and Partner Abuse, referring to perceived devaluation and relational mistreatment (e.g., ‘My partner devalues me’). Items are rated on a 5‐point Likert scale ranging from 1 (*not at all*) to 5 (*always*). Total scores range from 17 to 85, with higher scores indicating greater levels of PAD. In the original validation study, the scale demonstrated excellent internal consistency, with Cronbach's *α* = 0.89 for both the state and trait versions (Pugliese et al. 2025). In our study, it was 0.91 for the state version and 0.90 for the trait version.

Relational commitment was assessed using the Commitment subscale of the Investment Model Scale (IMS; Rusbult et al. 1998), a self‐report measure derived from the Investment Model of relationship commitment. The subscale assesses the individual's intent to persist in the relationship and their psychological attachment to the partner. The Commitment subscale consists of global items evaluating long‐term orientation and emotional involvement in the relationship (e.g., ‘I am committed to maintaining my relationship with my partner’; ‘I want our relationship to last for a very long time’). Items are rated on 9‐point Likert‐type scales ranging from 0 (*strongly disagree*) to 8 (*strongly agree*), and the total score ranges from 0 to 56, with higher scores indicating greater commitment to the relationship. In the original validation studies, the Commitment subscale demonstrated excellent internal consistency, with Cronbach's *α* values ranging from 0.91 to 0.95 across samples (Rusbult et al. 1998). In our study, it was 0.89.

Intention to return to the partner was assessed using the Intent to Return Questionnaire (ITRQ; Gordon et al. 2004), a brief self‐report measure designed to evaluate the extent to which women who have interrupted an abusive intimate relationship intend to reunite with their partner. The scale consists of five items assessing intentions and motivational aspects related to returning to the relationship. Items refer to thoughts, plans and desires concerning the resumption of the relationship (e.g., ‘I plan to see my partner again’; ‘I wish my partner and I could make our relationship work’). Responses are rated on a 5‐point Likert scale ranging from 1 (*strongly disagree*) to 5 (*strongly agree*), and the total score ranges from 1 to 25, with higher scores indicating a stronger intention to return to the partner. The ITRQ has demonstrated good internal consistency, with Cronbach's *α* values reported between 0.84 and 0.94 across studies (Gordon et al. 2004). In our study, it was 0.90.

Trauma‐related symptoms and associated functional impairment were assessed using the International Trauma Questionnaire (ITQ; Cloitre et al. 2018; Italian validation by Rossi et al. 2022), an 18‐item self‐report measure developed to assess PTSD and CPTSD in accordance with the ICD‐11 diagnostic criteria. The ITQ evaluates PTSD symptom clusters (e.g., ‘Have you had disturbing dreams that replay aspects of the experience or are clearly related to it?’) including re‐experiencing, avoidance and hyperarousal, as well as DSO (e.g., ‘When I am nervous, it takes me a long time to settle down’), comprising affective dysregulation, negative self‐concept and disturbances in relationships. Participants are asked to rate the severity of each symptom with reference to the past month, as well as the associated functional impairment. Items are scored on a 5‐point Likert scale ranging from 0 (*not at all*) to 4 (*extremely*), resulting in a total score including both symptom and functional impairment items, ranging from 0 to 72, with higher scores indicating greater trauma‐related symptoms and associated functional impairment. The ITQ has shown strong psychometric properties, with internal consistency coefficients ranging from 0.88 to 0.94 across PTSD and DSO subscales in the original validation studies (Cloitre et al. 2018) and a Cronbach's *α* of 0.88 for both subscales in the Italian validation (Rossi et al. 2022). In our study, it was 0.90.

General psychological well‐being was assessed using the Psychological General Well‐Being Index—Short form (PGWB‐S; Grossi et al. 2006), a brief six‐item self‐report measure designed to capture global subjective well‐being. Items are scored on a 6‐point Likert scale ranging from 0 (*none of the time*) to 5 (*all of the time*), yielding a total score between 0 and 30, with higher scores reflecting greater psychological well‐being. The PGWB‐S has demonstrated good to excellent internal consistency, with Cronbach's *α* values reported between 0.85 and 0.90 in validation studies (Grossi et al. 2006). Items assess both positive and negative aspects of emotional functioning, such as feelings of nervousness and positive affect (e.g., ‘Have you been bothered by nervousness or your “nerves” during the past month?’; ‘How often have you felt cheerful and light‐hearted?’). In our study, it was 0.85.

Resilience was assessed using the BRS (Smith et al. 2008), a six‐item self‐report measure specifically designed to assess resilience as the ability to bounce back or recover from stress. The scale is conceptualized as a unidimensional measure focusing on recovery from stressful events rather than on resilience‐related resources or protective factors. Items are rated on a 5‐point Likert scale ranging from 1 (*strongly disagree*) to 5 (*strongly agree*). Items 2, 4 and 6 were reverse scored before computing a total score by summing all six items (range 6–30). A summed score was used rather than the standard mean score to maintain consistency with the scoring approach adopted for the other self‐report measures included in the study. Example items include ‘I tend to bounce back quickly after hard times’ and ‘It is hard for me to snap back when something bad happens’. In the original validation study conducted across multiple samples, including university students and clinical populations, the BRS showed good internal consistency, with Cronbach's *α* values ranging from 0.80 to 0.91, and demonstrated a stable one‐factor structure (Smith et al. 2008). In our study, it was 0.90. All measures were administered at baseline (T0), after six sessions of imagery with rescripting (T1), at the end of treatment (T2) and at FU.

### 2.6 Statistical Analyses

Statistical analyses were conducted using JAMOVI (Version 2.7.17) statistical software and RStudio (R version 4.5.1). Descriptive statistics (means and standard deviations) were calculated for all outcome measures at each assessment time point. Because repeated baseline observations were not consistently available across baseline conditions, analyses of baseline level and trend changes were not feasible. Statistical analyses, therefore, focused on within‐subject change across the four predefined assessment points. To evaluate changes over time, repeated‐measures analyses of variance (RM‐ANOVAs) were performed separately for each primary and secondary outcome measure. Time was included as a within‐subject factor with four levels: baseline (T0), mid‐treatment (T1), post‐treatment (T2) and 1‐month FU. RM‐ANOVA was selected as a descriptive inferential approach to examine mean within‐subject change across repeated assessments; however, results were interpreted cautiously given the small sample size and uncontrolled design. When assumptions of sphericity were violated, Greenhouse–Geisser corrections were applied. Effect sizes were reported using partial eta squared (*η* ^2^p).

Following significant omnibus RM‐ANOVA results, post hoc pairwise comparisons between assessment time points were conducted in R using estimated marginal means. All possible pairwise comparisons among the four assessment time points were examined in order to characterize the timing and maintenance of within‐subject changes across the intervention and FU phases. These included baseline to mid‐treatment, baseline to post‐treatment, baseline to FU, mid‐treatment to post‐treatment, mid‐treatment to FU and post‐treatment to FU. Pairwise comparisons were adjusted using the Holm correction within each outcome measure across the six pairwise time comparisons, in order to control for Type I error inflation. Model‐based mean differences, standard errors, *t* ratios, Holm‐adjusted *p*‐values and unadjusted two‐sided 95% confidence intervals were reported for post hoc comparisons. Given the exploratory proof‐of‐concept nature of the study and the relatively small sample size, post hoc findings were interpreted cautiously and in conjunction with the omnibus RM‐ANOVA results and descriptive trends. To examine potential construct overlap among the primary relational outcomes, baseline correlations among state PAD, trait PAD, relational commitment and intention to return were also computed. All statistical tests were two‐tailed, and the level of statistical significance was set at *p* < 0.05. An a priori power analysis was conducted to guide recruitment for this exploratory proof‐of‐concept study. Because no prior trials had examined ImRs for this specific clinical target, the expected effect size was necessarily estimated from the broader ImRs literature (Morina et al. 2017). Previous studies and meta‐analytic evidence on ImRs interventions have reported large effects; however, we acknowledge that assuming a large effect size may be optimistic for a novel intervention targeting a novel construct. Therefore, the resulting sample size estimate should be interpreted as a pragmatic recruitment target for a preliminary feasibility and proof‐of‐concept study, rather than as a definitive indication of adequate power.

Assuming a large pre–post effect of *d* = 0.80, the analysis indicated that a minimum sample size of 15 participants would be required to achieve 80% statistical power, with *α* = 0.05, using a paired‐samples *t*‐test. Given the exploratory nature of the study and the small sample size, the findings should be interpreted cautiously and require replication in larger, adequately powered controlled trials using more conservative effect‐size assumptions.

### 2.7 Ethical Approval

The study was reviewed and approved by the Ethics Committee of the School of Cognitive Psychotherapy (Rome, Italy, Approval Code 2/24). All procedures were conducted in accordance with the ethical standards of the Declaration of Helsinki. Written informed consent was obtained from all participants prior to study enrolment. Participants were fully informed about the aims of the study, the voluntary nature of participation, their right to withdraw at any time without consequences and the procedures for data collection and handling. Given the vulnerability of the study population, specific measures were adopted to ensure participants' safety and confidentiality. Participants' identities were known only to the treating therapists and the referring anti‐violence centres. All data were anonymized using alphanumeric codes, and researchers involved in data analysis had no access to identifying information. Collaboration with anti‐violence centres ensured integrated support, including physical safety and legal assistance when needed.

## 3 Results

### 3.1 Sample Characteristics

All participants were women (*N* = 18; M age = 47.22 years, SD = 11.01). Most participants had experienced multiple forms of IPV and had ended the abusive relationship by baseline. Detailed sample characteristics are presented in Table 2.

**TABLE 2:** Sample characteristics.

Sociodemographic characteristics	
Age, years	47.22 (11.01)
Gender (female)	18 (100%)
IPV characteristics	
Psychological violence	18 (100%)
Physical violence	13 (72.2%)
Sexual violence	3 (16.7%)
Economic violence	4 (22.2%)
Stalking	1 (5.6%)
Multiple forms of victimization	**15 (83.3%)**
Relationship status at baseline	
No contact with the perpetrator	15 (83.3%)
Occasional contact with the perpetrator	2 (11.1%)
Still in the abusive relationship	1 (5.6%)

*Note:* Age is reported as mean (SD). All other values are reported as *n* (%). Participants could report more than one form of intimate partner violence; therefore, percentages for violence types do not sum to 100%.

### 3.2 Feasibility, Retention and Safety

Participant retention was excellent, with no dropouts during the intervention. No adverse events or clinically significant deterioration were reported to the first author or anti‐violence centre staff during the treatment period. However, adverse events were not assessed using a standardized checklist.

### 3.3 Descriptive Statistics

Descriptive statistics were computed for all primary and secondary outcome measures at each assessment time point. Means and standard deviations for the primary outcomes are reported in Table 3, whereas descriptive statistics for the secondary outcomes are presented in Table 4. Across measures, descriptive data showed systematic changes over time, with improvements from T0 to T2 and maintenance or slight attenuation of improvements at FU. Although the originally planned repeated‐baseline assessments were incomplete due to technical issues with the online questionnaire system, no missing data were observed for the final analytic repeated‐measures time points after treatment initiation, namely, baseline/pre‐treatment, mid‐treatment, post‐treatment and 1‐month FU. This absence of missing data refers only to the final analytic time points and not to the originally planned repeated‐baseline assessments.

**TABLE 3:** Means and standard deviations of primary outcome measures across assessment time points.

Measure	T0 mean (SD)	T1 mean (SD)	T2 mean (SD)	FU mean (SD)
PADS‐S	56.10 (13.90)	48.80 (14.40)	41.70 (11.00)	44.90 (14.20)
PADS‐T	53.50 (10.40)	48.40 (11.00)	39.40 (10.30)	42.10 (13.20)
RC	21.40 (10.49)	18.30 (9.70)	13.40 (9.70)	13.70 (10.70)
IR	9.06 (5.30)	6.72 (4.00)	5.44 (1.46)	5.78 (2.37)

Abbreviations: FU = 1‐month follow‐up; IR = intention to return; PADS‐S = Pathological Affective Dependence Scale—State version; PADS‐T = Pathological Affective Dependence Scale—Trait version; RC = relational commitment; T0 = baseline; T1 = after six sessions of imagery with rescripting; T2 = end of treatment.

**TABLE 4:** Means and standard deviations of secondary outcome measures across assessment time points.

Measure	T0 mean (SD)	T1 mean (SD)	T2 mean (SD)	FU mean (SD)
ITQ	41.40 (13.00)	33.10 (11.10)	22.60 (11.20)	28.30 (10.50)
PGWB	11.20 (5.07)	15.20 (4.82)	19.20 (5.48)	17.60 (4.29)
BRS	13.70 (5.07)	17.40 (4.64)	19.40 (4.13)	18.70 (4.14)

Abbreviations: BRS = Brief Resilience Scale; FU = 1‐month follow‐up; ITQ = International Trauma Questionnaire; PGWB = Psychological General Well‐Being; T0 = baseline; T1 = after six sessions of imagery with rescripting; T2 = end of treatment.

Table S1 presents the baseline correlation matrix among the primary relational outcomes, including state PAD, trait PAD, relational commitment and intention to return, to examine the degree of overlap among these related constructs. The correlations showed a significant positive association between state PAD and trait PAD (*r* = 0.497, *p* = 0.036) and between relational commitment and intention to return (*r* = 0.506, *p* = 0.032). In addition, trait PAD showed a moderate association with intention to return that approached statistical significance (*r* = 0.439, *p* = 0.068). Overall, this pattern suggests that the primary outcomes were related but not fully overlapping.

### 3.4 Primary Objectives

#### 3.4.1 Pathological Affective Dependence—State

An RM‐ANOVA revealed a significant main effect of time on PADS‐S scores across the four assessment points (T0–FU), *F*(3, 51) = 21.9, *p* < 0.001, *η* ^2^p = 0.56, indicating a large effect. Holm‐adjusted post hoc pairwise comparisons showed significant reductions in PADS‐S scores from baseline to mid‐treatment, from baseline to post‐treatment and from baseline to FU. A further significant reduction was observed from mid‐treatment to post‐treatment. In contrast, no significant differences emerged between mid‐treatment and FU or between post‐treatment and FU, suggesting that reductions were broadly maintained at FU without evidence of a statistically significant rebound.

#### 3.4.2 Pathological Affective Dependence—Trait

The RM‐ANOVA indicated a significant main effect of time on PADS‐T scores across all assessment points (T0–FU), *F*(3, 51) = 28.3, *p* < 0.001, *η* ^2^p = 0.62, indicating a large effect. Holm‐adjusted post hoc comparisons revealed significant decreases in PADS‐T scores from baseline to mid‐treatment, baseline to post‐treatment and baseline to FU. Significant reductions were also observed from mid‐treatment to post‐treatment and from mid‐treatment to FU. No significant difference emerged between post‐treatment and FU, indicating maintenance of treatment‐related reductions at FU.

#### 3.4.3 Relational Commitment

A significant main effect of time was found for RC across the four assessment points (T0–FU), *F*(3, 51) = 19.9, *p* < 0.001, *η* ^2^p = 0.53, indicating a large effect. Holm‐adjusted post hoc comparisons indicated significant reductions in relational commitment from baseline to mid‐treatment, baseline to post‐treatment and baseline to FU. Significant reductions were also observed from mid‐treatment to post‐treatment and from mid‐treatment to FU. No significant difference emerged between post‐treatment and FU, suggesting that reductions in relational commitment were maintained at FU.

#### 3.4.4 Intention to Return

**FIGURE 1:**
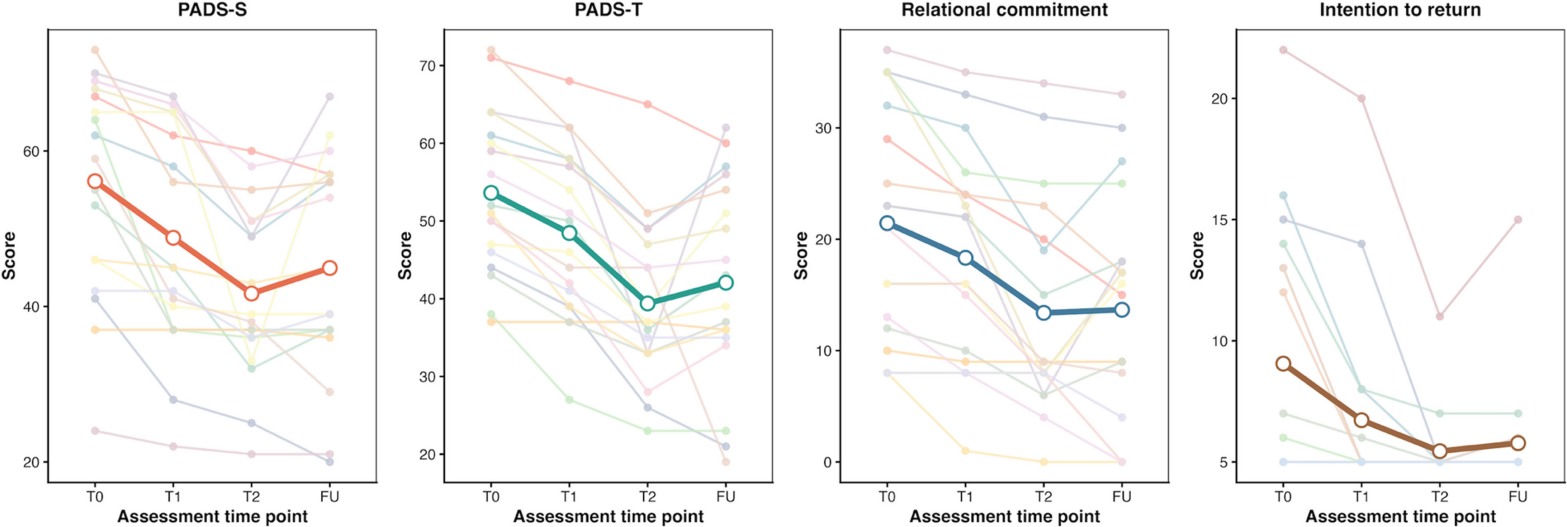
Individual trajectories for primary outcome measures across assessment time points. *Note:* Thin lines represent individual participant trajectories, and the thicker line represents the mean trajectory across participants. FU = 1‐month follow‐up; IR = intention to return; PADS‐S = Pathological Affective Dependence Scale—State version; PADS‐T = Pathological Affective Dependence Scale—Trait version; RC = relational commitment; T0 = baseline; T1 = after six imagery rescripting sessions; T2 = post‐treatment.

The RM‐ANOVA revealed a significant main effect of time on IR scores across assessment points (T0–FU), *F*(3, 51) = 9.26, *p* < 0.001, *η* ^2^p = 0.35, indicating a large effect. Holm‐adjusted post hoc comparisons showed significant reductions in intention to return from baseline to mid‐treatment, baseline to post‐treatment and baseline to FU. However, no significant differences emerged between mid‐treatment and post‐treatment, mid‐treatment and FU, or post‐treatment and FU. This suggests that the largest changes in intention to return occurred between baseline and subsequent assessment points, with reductions maintained over time. As an exploratory descriptive analysis, participants reporting any ongoing contact or involvement with the abusive partner at baseline (*n* = 3) showed higher intention to return scores than participants reporting no contact (M = 17.67 vs. 7.33). Their scores decreased over the course of treatment (post‐treatment: M = 7.00 vs. 5.13) and remained relatively low at FU (M = 8.67 vs. 5.20), although considerable variability was observed within this small subgroup. Visual inspection of individual trajectories (Figure 1) indicated that this variability was largely attributable to one participant, who also had the highest baseline PAD‐T score (71) and one of the highest baseline PAD‐S scores (67) in the sample. The other two participants showed trajectories broadly comparable to those of participants reporting no contact.

Post hoc pairwise comparisons were conducted as FU analyses to the omnibus RM‐ANOVAs using Holm adjustment within each outcome measure to control for Type I error inflation across the six pairwise comparisons. The results for the primary outcome measures are summarized in Table 5, whereas individual trajectories for the primary outcome measures are presented in Figure 1.

**TABLE 5:** Holm‐adjusted post hoc pairwise comparisons for primary outcome measures across assessment time points.

Outcome	Contrast	Estimate	SE	df	*t* ratio	*p* Holm‐adjusted	95% CI
PADS‐S	T0–T1	7.28	1.88	51	3.87	0.001	[3.50, 11.05]
	T0–T2	14.44	1.88	51	7.68	< 0.001	[10.67, 18.22]
	T0–FU	11.17	1.88	51	5.94	< 0.001	[7.39, 14.94]
	T1–T2	7.17	1.88	51	3.81	0.001	[3.39, 10.94]
	T1–FU	3.89	1.88	51	2.07	0.087	[0.12, 7.66]
	T2–FU	−3.28	1.88	51	−1.74	0.087	[−7.05, 0.50]
PADS‐T	T0–T1	5.17	1.70	51	3.03	0.008	[1.75, 8.59]
	T0–T2	14.22	1.70	51	8.35	< 0.001	[10.80, 17.64]
	T0–FU	11.56	1.70	51	6.78	< 0.001	[8.14, 14.98]
	T1–T2	9.06	1.70	51	5.32	< 0.001	[5.64, 12.48]
	T1–FU	6.39	1.70	51	3.75	0.001	[2.97, 9.81]
	T2–FU	−2.67	1.70	51	−1.57	0.124	[−6.09, 0.75]
RC	T0–T1	3.11	1.23	51	2.52	0.029	[0.64, 5.59]
	T0–T2	8.06	1.23	51	6.54	< 0.001	[5.58, 10.53]
	T0–FU	7.78	1.23	51	6.31	< 0.001	[5.30, 10.25]
	T1–T2	4.94	1.23	51	4.01	< 0.001	[2.47, 7.42]
	T1–FU	4.67	1.23	51	3.79	0.001	[2.19, 7.14]
	T2–FU	−0.28	1.23	51	−0.23	0.823	[−2.75, 2.20]
IR	T0–T1	2.33	0.76	51	3.08	0.013	[0.81, 3.85]
	T0–T2	3.61	0.76	51	4.77	< 0.001	[2.09, 5.13]
	T0–FU	3.28	0.76	51	4.33	< 0.001	[1.76, 4.80]
	T1–T2	1.28	0.76	51	1.69	0.293	[−0.24, 2.80]
	T1–FU	0.94	0.76	51	1.25	0.436	[−0.58, 2.46]
	T2–FU	−0.33	0.76	51	−0.44	0.662	[−1.85, 1.19]

*Note:* Estimates represent model‐based mean differences between assessment time points. Positive estimates indicate higher scores at the first time point relative to the second time point. *p*‐values are two‐tailed and were adjusted using the Holm correction within each outcome measure across the six pairwise comparisons. Confidence intervals are unadjusted two‐sided 95% confidence intervals.

Abbreviations: CI = confidence interval; FU = 1‐month follow‐up; IR = intention to return; PADS‐S = Pathological Affective Dependence Scale—State version; PADS‐T = Pathological Affective Dependence Scale—Trait version; RC = relational commitment; T0 = baseline; T1 = after six imagery rescripting sessions; T2 = post‐treatment.

### 3.5 Secondary Objectives

#### 3.5.1 Post‐Traumatic Stress Symptoms

An RM‐ANOVA showed a significant main effect of time on ITQ scores across the four assessment points (T0–FU), *F*(3, 51) = 52.4, *p* < 0.001, *η* ^2^p = 0.75, indicating a large effect. Holm‐adjusted post hoc pairwise comparisons indicated significant reductions in ITQ scores from baseline to mid‐treatment, baseline to post‐treatment and baseline to FU. Significant reductions were also observed from mid‐treatment to post‐treatment and from mid‐treatment to FU. However, ITQ scores significantly increased from post‐treatment to FU, indicating a partial symptom rebound, although FU scores remained lower than baseline levels.

#### 3.5.2 Psychological Well‐Being

The RM‐ANOVA revealed a significant main effect of time on PGWB scores across assessment points (T0–FU), *F*(3, 51) = 34.1, *p* < 0.001, *η* ^2^p = 0.66, indicating a large effect. Holm‐adjusted post hoc comparisons showed significant increases in psychological well‐being from baseline to mid‐treatment, baseline to post‐treatment and baseline to FU. Significant increases were also observed from mid‐treatment to post‐treatment and from mid‐treatment to FU. The decrease from post‐treatment to FU did not reach statistical significance after Holm correction, suggesting a non‐significant attenuation of well‐being levels after the end of treatment.

#### 3.5.3 Resilience

A significant main effect of time was found for BRS scores across the four assessment points (T0–FU), *F*(3, 51) = 25.8, *p* < 0.001, *η* ^2^p = 0.60, indicating a large effect. Holm‐adjusted post hoc comparisons indicated significant increases in resilience from baseline to mid‐treatment, baseline to post‐treatment and baseline to FU. A further significant increase was observed from mid‐treatment to post‐treatment. No significant differences emerged between mid‐treatment and FU or between post‐treatment and FU, suggesting that improvements in resilience were broadly maintained at FU.

**FIGURE 2:**
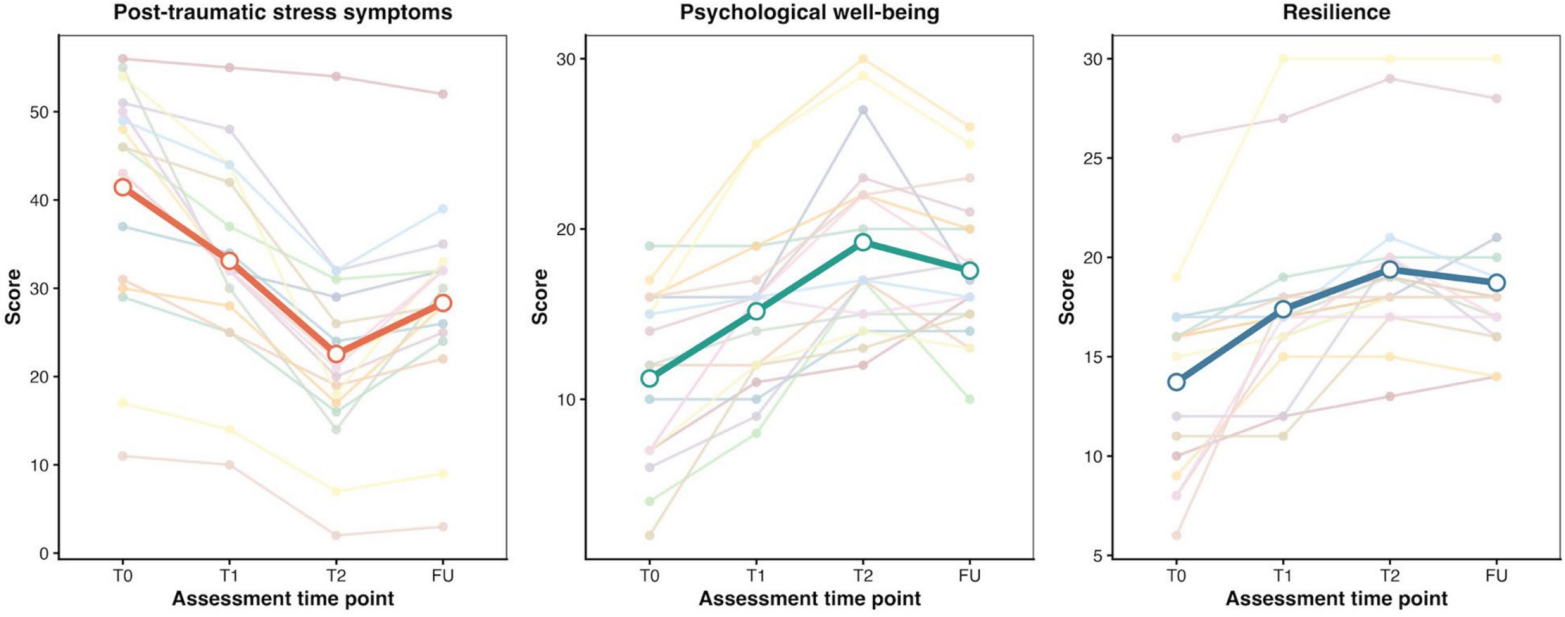
Individual trajectories for secondary outcome measures across assessment time points. *Note:* Thin lines represent individual participant trajectories, and the thicker line represents the mean trajectory across participants. BRS = Brief Resilience Scale; FU = 1‐month follow‐up; ITQ = International Trauma Questionnaire; PGWB = Psychological General Well‐Being; T0 = baseline; T1 = after six imagery rescripting sessions; T2 = post‐treatment.

As for the primary outcomes, post hoc pairwise comparisons for secondary outcomes were conducted as FU analyses to the omnibus RM‐ANOVAs using Holm adjustment within each outcome measure to control for Type I error inflation across the six pairwise comparisons. The results for the secondary outcome measures are summarized in Table 6, whereas individual trajectories for the secondary outcome measures are presented in Figure 2.

**TABLE 6:** Holm‐adjusted post hoc pairwise comparisons for secondary outcome measures across assessment time points.

Outcome	Contrast	Estimate	SE	df	*t* ratio	*p* Holm‐adjusted	95% CI
ITQ	T0–T1	8.33	1.56	51	5.34	< 0.001	[5.20, 11.47]
	T0–T2	18.89	1.56	51	12.11	< 0.001	[15.76, 22.02]
	T0–FU	13.11	1.56	51	8.40	< 0.001	[9.98, 16.24]
	T1–T2	10.56	1.56	51	6.76	< 0.001	[7.42, 13.69]
	T1–FU	4.78	1.56	51	3.06	0.004	[1.65, 7.91]
	T2–FU	−5.78	1.56	51	−3.70	0.001	[−8.91, −2.65]
PGWB	T0–T1	−3.94	0.84	51	−4.69	< 0.001	[−5.63, −2.26]
	T0–T2	−8.00	0.84	51	−9.51	< 0.001	[−9.69, −6.31]
	T0–FU	−6.33	0.84	51	−7.53	< 0.001	[−8.02, −4.65]
	T1–T2	−4.06	0.84	51	−4.82	< 0.001	[−5.74, −2.37]
	T1–FU	−2.39	0.84	51	−2.84	0.013	[−4.08, −0.70]
	T2–FU	1.67	0.84	51	1.98	0.053	[−0.02, 3.35]
BRS	T0–T1	−3.67	0.70	51	−5.21	< 0.001	[−5.08, −2.25]
	T0–T2	−5.67	0.70	51	−8.05	< 0.001	[−7.08, −4.25]
	T0–FU	−5.00	0.70	51	−7.10	< 0.001	[−6.41, −3.59]
	T1–T2	−2.00	0.70	51	−2.84	0.019	[−3.41, −0.59]
	T1–FU	−1.33	0.70	51	−1.89	0.128	[−2.75, 0.08]
	T2–FU	0.67	0.70	51	0.95	0.348	[−0.75, 2.08]

*Note:* Estimates represent model‐based mean differences between assessment time points. Positive estimates indicate higher scores at the first time point relative to the second time point. *p*‐values were two‐tailed and adjusted using the Holm correction within each outcome measure across the six pairwise comparisons. Confidence intervals are unadjusted two‐sided 95% confidence intervals.

Abbreviations: BRS = Brief Resilience Scale; CI = confidence interval; FU = 1‐month follow‐up; ITQ = International Trauma Questionnaire; PGWB = Psychological General Well‐Being; T0 = baseline; T1 = after six imagery rescripting sessions; T2 = post‐treatment.

Although average changes over time were observed across several outcomes, response patterns appeared heterogeneous across participants, suggesting variability in the magnitude and maintenance of treatment‐related changes at the individual level.

## 4 Discussion

The present exploratory single‐arm repeated‐measures study provides preliminary evidence on ImRs‐PAD, an intervention designed to address trauma‐related relational vulnerability in IPV survivors. Developed within the PAD framework, ImRs‐PAD addresses chronic unmet needs for love, dignity and safety that underlie affective dependence and relational entrapment. The results showed significant reductions in both state‐level and trait‐level PAD across the intervention period. State‐level PAD decreased significantly from baseline to mid‐treatment, post‐treatment and FU, with a further significant reduction from mid‐treatment to post‐treatment. This pattern suggests short‐term reductions in trauma‐related relational vulnerability that may be clinically relevant to reducing difficulties in disengaging from harmful relationships. Although mean scores showed a slight descriptive increase from post‐treatment to FU, this change was not statistically significant after Holm correction, suggesting that reductions in state‐level PAD were broadly maintained at FU. Trait‐level PAD, which reflects more stable patterns of PAD rooted in long‐standing relational schemas (Pugliese et al. 2025), also declined significantly over the course of the intervention. These reductions were maintained at FU, with no evidence of a statistically significant rebound.

These findings extend a previous online multiple case series study, in which only state‐level PAD had been assessed (Pugliese et al. 2026), by suggesting that changes may also occur across related relational indicators, including trait PAD, relational commitment and intention to return. For instance, relational commitment decreased significantly from baseline to mid‐treatment, post‐treatment and FU. Further reductions were observed from mid‐treatment to post‐treatment and to FU, whereas no significant difference emerged between post‐treatment and FU, suggesting that reductions in relational commitment were maintained after treatment termination. Because relational commitment reflects processes that, while generally adaptive, may become maladaptive in the context of violent relationships—particularly in terms of difficulties in emotional disengagement (Rusbult et al. 1998)—these results are clinically relevant. They are consistent with the proposed mechanism of change, whereby ImRs‐PAD modifies maladaptive attachment‐related representations that contribute to relational entrapment and reinforce affective dependence. Finally, the intention to return to the abusive partner declined significantly from baseline to all subsequent assessment points, with no significant differences between mid‐treatment, post‐treatment and FU. This pattern suggests that the largest change in intention to return occurred between baseline and the subsequent assessment points and that this reduction was maintained over time. This result carries potential clinical relevance from a safety perspective, as returning to an abusive relationship is associated with heightened risk of violence escalation, serious injury and femicide (Bell et al. 2007; Gordon et al. 2004). However, because actual return behaviours, revictimization events and safety outcomes were not directly assessed, this finding should be interpreted cautiously. The observed decrease in intention to return may indicate a change in motivational orientation towards the abusive relationship, rather than direct evidence of protective behavioural change or reduced revictimization risk. Given the conceptual proximity among the primary relational outcomes, PAD state, PAD trait, relational commitment and intention to return should not be interpreted as fully independent domains of change. Rather, they may represent conceptually related indicators of IPV‐related relational entrapment. To address this issue, we examined baseline correlations among these outcomes. The pattern of correlations suggested that these constructs were related but not redundant. In particular, state and trait PAD appeared to capture connected aspects of the same underlying relational vulnerability, whereas relational commitment and intention to return seemed to reflect a more explicit relationship‐maintenance domain. Importantly, PAD does not simply assess a conscious wish to remain in, or return to, the abusive relationship but captures a broader pattern of partner abuse, internal conflict and perceived inability to separate. Therefore, the present findings should be interpreted as preliminary changes across conceptually related but distinct indicators of relational entrapment, rather than as evidence that ImRs‐PAD affected several fully independent domains.

This interpretation is also consistent with the clinical phase of the present sample. Participants had already entered a pathway with an anti‐violence centre and had reported the abuse, suggesting that they had taken important cognitive, legal or behavioural steps towards protection and disengagement from the abusive relationship. In this context, explicit endorsement of commitment or intention to return may be low because survivors consciously recognize the danger of the relationship and are actively working towards safety. However, explicitly reporting low commitment or low intention to return does not necessarily imply the absence of PAD‐related vulnerability. Survivors may state that they do not intend to return to the abusive partner, while still experiencing an implicit emotional pull towards the bond. PAD may therefore persist as an internal conflict between the reflective intention to remain safe and the emotional mechanisms that sustain relational entrapment, including guilt, fear of abandonment, excessive responsibility for the partner and perceived inability to fully detach. This interpretation is further supported by the observation that, among the three participants who maintained contact with the perpetrator during the treatment period, the participant who showed persistently higher intention to return scores across the study also displayed the most severe PAD profile. Although based on a single case, this pattern is consistent with the view that elevated intention to return may reflect a broader profile of relational vulnerability, rather than simply the presence of ongoing contact with the abusive partner.

Future studies with adequately powered samples should further examine the degree of construct overlap among these variables and clarify whether changes in PAD precede or mediate changes in relational commitment and intention to return to an abusive partner.

With respect to trauma symptoms, participants showed marked reductions in CPTSD symptoms from baseline to post‐treatment. At FU, ITQ scores increased from post‐treatment to FU, indicating a partial symptom rebound, although FU scores remained substantially lower than baseline levels. This pattern suggests that symptom improvement was not fully stable after treatment termination and differs from findings reported in some previous applications of ImRs in trauma‐exposed populations, where symptom reductions were more consistently maintained over time (Daniëls et al. 2025; Pugliese et al. 2026; Visco‐Comandini et al. 2025). Psychological well‐being and resilience showed significant increases during the intervention, indicating improvements that extend beyond symptom reduction and reflect broader processes of psychological recovery. Psychological well‐being showed a non‐significant attenuation from post‐treatment to FU after Holm correction, whereas resilience improvements were broadly maintained at FU. This is consistent with research showing that ImRs is associated not only with reductions in PTSD symptoms but also with significant improvements in adaptive psychological functioning, such as adaptive schema modes (De Haan et al. 2020), as well as with sustained benefits on mental health outcomes at FU (Kip et al. 2023). Together, these findings suggest that ImRs‐PAD may foster adaptive functioning and psychological resources relevant to recovery, although the partial rebound in trauma symptoms highlights the importance of continued support after treatment.

### 4.1 Contextual and Systemic Factors Influencing Post‐Treatment Outcomes

The partial symptom rebound observed at FU may be interpreted as potentially consistent with the influence of ongoing contextual stressors commonly faced by IPV survivors. After treatment ends, many remain exposed to relational and environmental threats, including contact with the abusive partner due to shared parenting, economic dependence or legal proceedings (Spearman et al. 2023). In the absence of sustained therapeutic or institutional support, these unresolved stressors may function as trauma reminders and lead to symptom reactivation—even when core relational mechanisms have improved. This interpretation aligns with Fernández‐Fillol et al. (2021), who describe such fluctuations as context sensitive rather than indicative of treatment failure.

The service context in which the intervention was delivered may also have contributed to this pattern. In contrast to an earlier online multiple case series (Pugliese et al. 2026), where participants maintained post‐treatment contact, at the end of this intervention, participants did not receive any further therapeutic support. The therapists—clinical trainees delivering the protocol—left the service after completing their internship, resulting in the closure of the therapy and the termination of contact. More broadly, evidence suggests that psychosocial interventions for IPV survivors may have limited long‐term maintenance, whereas more intensive and integrative interventions combining psychological and advocacy‐based components appear to be more favourable (Micklitz et al. 2024). Nevertheless, as post‐treatment support was not experimentally manipulated, this explanation should be considered hypothesis generating.

The sociocultural environment may represent another relevant contextual factor (Guidi et al. 2024). Most participants anecdotally described their local environment as marked by restrictive gender norms, conservative social expectations and limited institutional responsiveness to IPV, which they perceived as significantly shaping both their experiences of violence and their ability to seek help. Cross‐national studies highlight how cultural frameworks and levels of gender equality shape perceptions of IPV and engagement with services (Hayes and Pinchevsky 2023; Karlsson et al. 2022). However, because sociocultural variables were not formally assessed in the present study, these considerations should be interpreted as contextual observations rather than empirical findings. Future studies should directly measure sociocultural and service‐related variables to examine how they may moderate treatment response and post‐treatment maintenance.

### 4.2 Clinical Implications

The present findings provide preliminary support for the use of ImRs as an intervention targeting relational trauma vulnerability that may be involved in exposure to IPV. Reductions in PAD, RC and IR to the abusive partner suggest shifts in key relational dimensions that may be clinically relevant to emotional disengagement from abusive relationships and, potentially, to longer term safety, as continued relational involvement may prolong exposure to recurrent violence and serious harm (Bell et al. 2007). However, these clinical implications should be considered preliminary and limited to short‐term changes observed over the intervention period and 1‐month FU. Given the brevity of FU, the present findings cannot establish whether reductions in PAD, relational commitment or intention to return translate into stable relational disengagement over time. Longer FU assessments are needed to evaluate whether these changes are maintained in the context of recurrent relational instability, possible ongoing contact with the abusive partner and risk of re‐engagement. Moreover, because behavioural outcomes, actual revictimization events and safety indicators were not directly measured, these findings should not be interpreted as evidence that ImRs‐PAD reduces recurrent violence or femicide risk. Rather, they suggest a potential change in psychological mechanisms that may be relevant to future safety‐oriented outcomes and should be tested in larger controlled studies. The partial rebound observed at FU highlights the importance of embedding ImRs‐based interventions within broader systems of ongoing support. Clinically, this suggests that ImRs‐PAD may be most effective when delivered as part of an integrated care pathway that includes continued therapeutic contact, access to anti‐violence services and practical and social support. In terms of feasibility, the absence of dropout and the consistent engagement observed across sessions provide preliminary evidence that the intervention was feasible to deliver and complete within this clinical context, despite the clinical complexity of the sample. Acceptability, however, was not directly assessed and should be evaluated in future studies using participant feedback, satisfaction measures or other dedicated indicators. Finally, the feasibility of delivering ImRs‐PAD by early‐career therapists under supervision supports its potential implementation within routine clinical and anti‐violence services. At the same time, its implementation may require appropriate training and organizational support, which should be considered when evaluating its scalability in different service contexts. This scalability is particularly important in high‐demand settings with limited specialist resources.

### 4.3 Limitations and Future Directions

The present findings should be interpreted with caution. First, although the original protocol envisioned a multiple‐baseline design to enhance internal validity, technical difficulties during early data collection (specifically, a failure in the online survey platform) prevented the collection of repeated baseline measurements for several participants. As a result, while a single baseline assessment was available, the planned prolonged baseline phase could not be implemented consistently across cases. Therefore, the study was conducted as an exploratory single‐arm repeated‐measures proof‐of‐concept study, with assessments administered at fixed time points. While this approach limits causal inference, it remains appropriate for examining within‐subject change over time in preliminary clinical research. Accordingly, the present findings should be interpreted as exploratory proof‐of‐concept evidence rather than as evidence of treatment efficacy or causal effects. Future studies using controlled designs and stronger methodological controls are needed to determine whether the observed changes can be attributed to the intervention itself. Second, the small sample size and the absence of a control group further limit the strength of causal conclusions. In addition, non‐specific therapeutic factors—such as therapeutic alliance, support from the anti‐violence centre, psychoeducation, repeated assessments and expectations of benefit—may have contributed to the observed improvements. Because these factors were not formally measured or controlled, future controlled studies should include comparison conditions and process measures to disentangle specific ImRs‐PAD mechanisms from common therapeutic factors. For the same reason, the large within‐subject effect sizes observed in this study should not be interpreted as evidence of treatment‐specific efficacy. In the absence of a control group, these estimates may be inflated by non‐specific therapeutic factors, repeated assessment, regression to the mean or contextual changes and should therefore be considered preliminary signals of change requiring replication in controlled studies.

Moreover, although intervention fidelity and adherence were supported through a structured protocol and regular supervision, they were not formally assessed through independent ratings, session recordings or standardized adherence checklists. Therefore, the degree of protocol fidelity cannot be fully established and should be evaluated more systematically in future studies.

Third, the FU period was limited to 1 month and occurred without continued therapeutic contact, which may have contributed to the partial rebound in symptomatology observed post‐treatment. This short FU window is a relevant limitation, as it does not allow conclusions about the long‐term stability of changes in IPV‐related relational processes such as PAD, relational commitment and intention to return. Fourth, the study relied exclusively on self‐report measures, which may be influenced by response biases, social desirability, limited insight or changes in willingness to disclose sensitive experiences. Fifth, relevant contextual variables, including duration of the abusive relationship, time since separation and socio‐economic characteristics, were not systematically assessed and should be included in future studies to better understand treatment response and long‐term outcomes. Future studies with larger and adequately powered samples should also examine whether psychological variables—such as attachment insecurity, emotion‐regulation difficulties, dissociation, self‐compassion and perceived self‐efficacy—predict, mediate or moderate short‐, medium‐ and long‐term changes in PAD and broader clinical outcomes. Where feasible, assessments should include clinician‐rated measures, qualitative interviews, biological indicators of stress‐related dysregulation and behavioural or service‐use indicators. Future trials could also evaluate whether booster sessions or structured aftercare enhance the durability of treatment‐related changes.

## 5 Conclusion

The present exploratory proof‐of‐concept study offers preliminary evidence that ImRs‐PAD may be a promising intervention for addressing trauma‐related relational vulnerability in survivors of IPV. Participant retention was excellent, supporting the preliminary feasibility of the intervention within real‐world anti‐violence services, including delivery by supervised trainees in this clinical context. Participation in the intervention was associated with changes in PAD, relational outcomes relevant to disengagement, trauma‐related symptoms and broader indicators of psychological recovery. Changes in the primary relational outcomes were more consistently maintained at FU than improvements in trauma‐related symptoms, which showed a partial rebound after treatment termination but remained below baseline levels. These findings highlight the importance of sustained support beyond treatment. Further controlled studies are needed to assess the replicability, generalizability and long‐term impact of these preliminary findings.

## Author Contributions

The ImRs‐PAD intervention was independently conceptualized and developed by Erica Pugliese. **Erica Pugliese:** conceptualization, methodology, writing – original draft, writing – review and editing, supervision. **Allison Uvelli:** methodology, data curation, formal analysis, writing – original draft. **Maria Grazia Foschino Barbaro:** resources, supervision. **Carolina Papa:** methodology, data curation, formal analysis, writing – review and editing. **Federica Visco‐Comandini:** investigation, validation. **Francesco Mancini:** project administration. **Arnold A. P. van Emmerik:** writing – review and editing, supervision.

## Ethics Statement

This study was approved by the Ethics Committee of the School of Cognitive Psychotherapy (Rome, Italy, Protocol No. 2/24).

## Conflicts of Interest

Erica Pugliese occasionally offers training in ImRs‐PAD. Her remuneration for training activities partially supports her ongoing research. The other authors declare no conflicts of interest.

## Use of Artificial Intelligence

The authors used ChatGPT (OpenAI) to support language editing. All content was reviewed and approved by the authors, who take full responsibility for the final version of the manuscript.

## Supporting information


**Table S1:** Baseline correlation matrix for primary outcomes.

## Data Availability

The data that support the findings of this study are not publicly available due to ethical and privacy restrictions related to the sensitive nature of the data (i.e., intimate partner violence). Anonymized data may be made available from the corresponding author upon reasonable request and subject to approval by the relevant ethics committee.

## Appendix A ImRs‐PAD Intervention Protocol

The following appendix provides a detailed outline of the manualized ImRs‐PAD protocol used in the present study. The same protocol has been described elsewhere in an online delivery format (Pugliese et al. 2026); in the present study, it was delivered in person to IPV survivors.

The ImRs‐PAD intervention consisted of 13 weekly individual sessions of approximately 50 min each. The first session focused on providing psychoeducation about PAD and introducing the ImRs‐PAD protocol. The remaining 12 sessions involved active ImRs treatment.

The protocol was organized into three sequential phases following a past–present–future framework. The intervention targeted unmet core emotional needs, particularly needs for love, dignity and safety, within early family experiences, current or recent abusive partner relationships and anticipated future relational situations. In this framework, love does not simply refer to romantic affection but to the basic emotional experience of being worthy of care, being allowed to prioritize oneself and not having to organize one's identity around the excessive caregiving of a fragile, problematic or abusive other. The therapist initially took an active role in guiding the rescripting process, modelling protective and validating responses, providing emotional containment and helping the patient access unmet emotional needs. As treatment progressed, patients were gradually encouraged to assume a more active role in rescripting their experiences, thereby promoting autonomy, affect regulation, self‐protection and relational agency.

### Phase 1: Childhood Adverse Memories and Unmet Emotional Needs

Sessions 2–7 focused on emotionally significant childhood memories involving caregivers and early relational experiences associated with the chronic frustration of core emotional needs, particularly love, dignity and safety. Starting from current PAD‐related difficulties, such as fear of abandonment, perceived inability to separate, excessive responsibility for the partner or difficulty prioritizing oneself, patients were guided to identify earlier autobiographical memories in which similar emotional meanings were first encoded. For example, a patient who felt unable to leave an abusive partner because she experienced the partner as fragile, dependent or unable to survive without her might be guided to an early memory in which she had to take care of an emotionally unavailable, vulnerable or dysregulated caregiver. During rescripting, the therapist intervened in the image to protect the child, validate her emotional experience, remove inappropriate responsibility and provide the care, recognition and safety that were missing at the time. The aim was to transform meanings such as ‘I am responsible for the other’, ‘my needs do not matter’, ‘I am not worthy of protection’ or ‘I cannot be safe if I choose myself’.

### Phase 2: Traumatic and Emotionally Significant Memories Involving the Current or Former Partner

Sessions 8–10 focused on traumatic or emotionally significant memories involving the current or former abusive partner. These memories were selected because they were linked to PAD‐specific relational mechanisms, such as helplessness, unworthiness, fear of abandonment, shame, excessive responsibility for the partner, difficulty setting boundaries and perceived inability to separate. For example, a patient might rescript a memory in which she remained silent during an episode of psychological abuse because she feared abandonment, retaliation or the partner's emotional collapse. During the rescripting procedure, the therapist helped the patient imagine receiving protection, validation and support while also promoting new responses such as asserting herself, setting boundaries, leaving the situation, contacting supportive others or prioritizing her own safety and dignity. The aim was not only to reduce distress associated with the traumatic memory but also to modify the relational meanings that maintained affective entrapment.

### Phase 3: Future‐Oriented Imagery, Self‐Protection and Relapse Prevention

Sessions 11–13 focused on future‐oriented imagery exercises aimed at strengthening self‐protection, separation‐related mastery and relapse prevention. Patients rehearsed adaptive responses to anticipated interpersonal challenges that could reactivate PAD‐related mechanisms.

For example, survivors might rehearse responding to situations in which an abusive partner asks for forgiveness, promises to change, threatens suicide, minimizes the abuse, blames the victim or attempts to re‐establish contact. Imagery procedures focused on helping the patient recognize emotional triggers, tolerate guilt or fear of abandonment, maintain boundaries, seek support and prioritize her own safety, dignity and emotional needs. These exercises aimed to consolidate a new relational position in which the patient could experience herself as worthy of protection and no longer responsible for preserving the relationship at the cost of herself.
