# Supplementary material for: Imagery Rescripting for Pathological Affective Dependence in Intimate Partner Violence (ImRs‐PAD): An In‐Person Proof‐of‐Concept Study
Source: Clin Psychol Psychother. 2026 Sep 24;33(5):e70339. doi: 10.1002/cpp.70339 (PMC13613076; doi:10.1002/cpp.70339)
Supplement: Supplementary file 1 [file CPP-33-e70339-s001.docx]

**Table S1**

*Baseline Correlation Matrix for Primary Outcomes*

|  | **PADS-S** | **PADS-T** | **RC** | **IR** |
| --- | --- | --- | --- | --- |
| **PADS-S** | - |  |  |  |
| **PADS-T** | .497* | - |  |  |
| **RC** | .058 | .296 | - |  |
| **IR** | .165 | .439 | .506* | - |

*Note.* Values are Pearson’s correlation coefficients. PADS-S = Pathological Affective Dependence Scale–State; PADS-T = Pathological Affective Dependence Scale–Trait; RC = Relational Commitment; IR = Intention to Return. p < .05.
